# Delayed Intraperitoneal Catheter Erosion into the Small Bowel

**DOI:** 10.1155/2015/697608

**Published:** 2015-05-06

**Authors:** Lauren Kerwin, Sean Calhoun

**Affiliations:** Morristown Medical Center, Morristown, NJ 07960, USA

## Abstract

Intraperitoneal chemotherapy can be provided in cases of metastatic ovarian carcinoma. Although most complications arise during or immediately after insertion of the catheter, there are complications that can arise several months later or during therapy administration. One of these delayed complications is catheter erosion into adjacent bowel.

## 1. Introduction

Intraperitoneal chemotherapy can be provided in cases of metastatic ovarian carcinoma. Although most complications arise during or immediately after insertion of the catheter, there are complications that can arise several months later or during therapy administration. One of these delayed complications is catheter erosion into adjacent bowel. Several modalities can be utilized to confirm these findings including CT of the abdomen and pelvis. Interventional radiology can also be utilized to confirm the location of the catheter in question. Evaluating for complications is imperative for prompt and appropriate treatment and so patients can receive additional chemotherapy therapy as needed.

## 2. Case Report

A 61-year-old woman with no significant past medical history was diagnosed with ovarian cancer in February of 2009. The patient was treated with IV chemotherapy prior to her TAH-BSO performed in November of 2010.

On April 30, 2012, the patient returned with abdominal discomfort and a repeat CT scan on May 9, 2012, demonstrated omental caking around the liver, left splenic flexure, and a large mass in the left lower abdomen invading the descending colon consistent with recurrent disease.

The patient returned to the operating room on May 17, 2012. A left hemicolectomy was performed. No other residual disease was identified and a Bard cuffless intraperitoneal (IP) catheter was placed for IP chemotherapy treatment.

**Figure 1:**
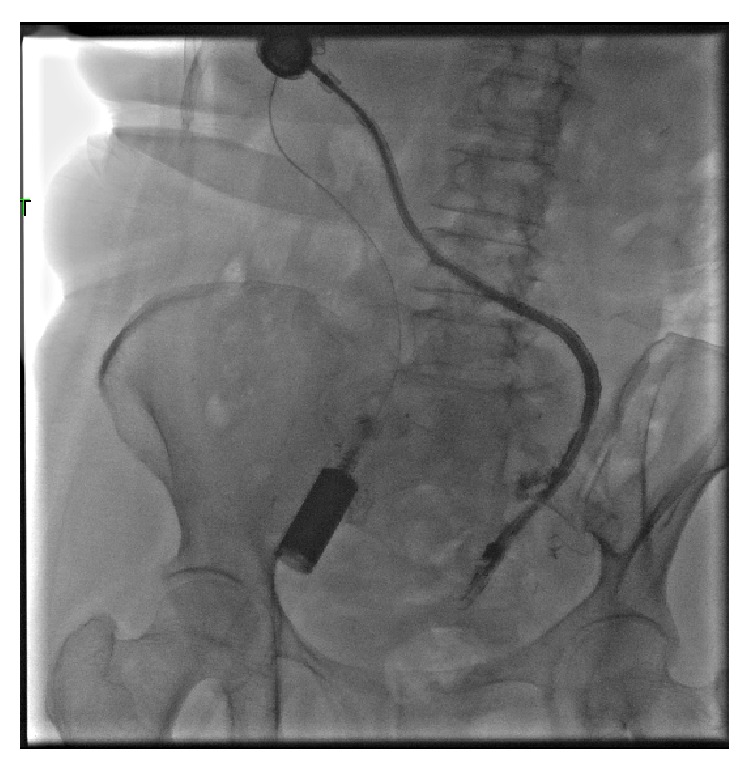
Initially, a fluoroscopic examination of the peritoneal catheter was performed because the catheter was not functioning well. The images show contrast flowing around the catheter with little spillage into the pelvis, consistent with a fibrin sheath.

**Figure 2:**
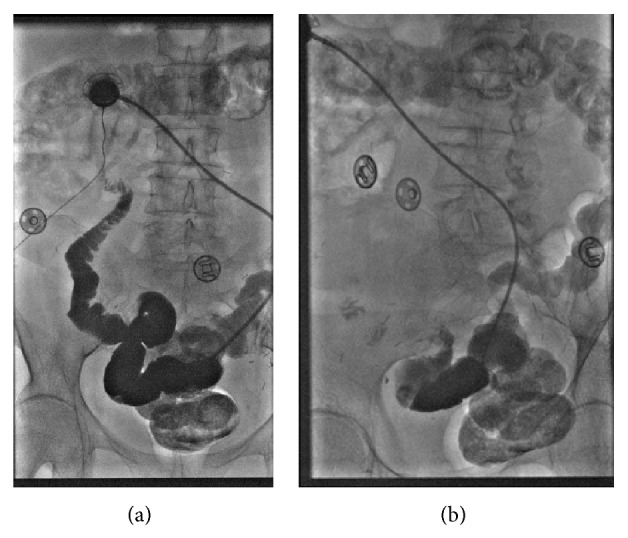
Several fluoroscopic images provided approximately one month later show contrast being injected into the port and now entering several loops of small bowel, consistent with erosion into the bowel lumen.

**Figure 3:**
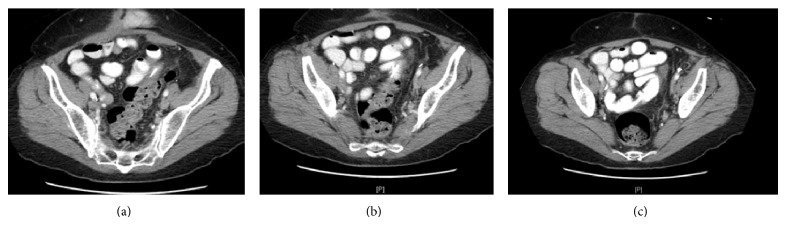
Serial images from a CT of the abdomen and pelvis with IV and oral contrast show the peritoneal catheter in the lower pelvis. The catheter approaches and then enters a loop of small bowel, confirming the position of the catheter as seen in the prior fluoroscopic images.

The patient underwent three complete cycles of IP chemotherapy. Halfway through the fourth cycle, the catheter began to malfunction and the patient experienced abdominal discomfort. On August 28, 2012, a fluoroscopic study of the catheter was performed demonstrating a fibrin sheath around the distal catheter tip with little spillage and distribution into the pelvis (Figure 1). The patient returned approximately one month later complaining of continuous problems with the catheter. A subsequent fluoroscopic study was performed, showing contrast entering multiple small bowel loops, concerning for bowel perforation (Figure 2). A CT scan was performed showing the catheter possibly eroding into the small bowel. The patient returned to the OR. Evaluation of a loop of bowel in the left lower quadrant demonstrated a 1-2 cm defect in the wall, likely representing the area where the tubing had eroded into the wall and this portion of the small bowel was resected (Figure 3).

## 3. Discussion

Several studies have been performed comparing intravenous (IV) chemotherapy with intraperitoneal (IP) chemotherapy and have suggested several advantages of IP therapy over IV including greater concentrations of therapeutic drug in the region of interest, increased peritoneal exposure, and fewer systemic side effects [1].

Complications with intraperitoneal catheters are not uncommon. In a study by Makhija et al., 10% of patients experienced complications from malfunction or infection [1]. However, these complications usually occur during insertion of the catheter, immediately after insertion, or during the administration of chemotherapy. There are few cases where complications involving the catheter are discovered several months after catheter insertion or after several rounds of IP chemotherapy. A few of these cases of delayed catheter complications have involved erosion and perforation into viscera including the rectum, the bladder with extension into the external urethral meatus, and the vagina causing an enterovaginal fistula [2, 3].

Delayed erosion into the bowel is uncommon. In previous studies, bowel perforation has been shown to be infrequent. Piccart et al. reported bowel perforation 2.4% of the time, Braly et al. 3%, and Davidson et al. 3.5% [4]. Several studies have suggested that placing the catheter while simultaneously undergoing laparotomy and bowel resection predisposes to catheter erosion into the bowel [4]. Another factor that has been associated with bowel erosion and perforation has been the use of a fenestrated versus a nonfenestrated catheter.

Varney et al. [5] reported a case of catheter-enteric fistula. After the patient had a TAH for ovarian carcinoma, she had a separate procedure a month later for the insertion of a Tenckhoff catheter, where the small bowel was accidentally perforated and repaired during surgery. Following successful cycles of chemotherapy, the patient presented with fever and abdominal pain and a fistula was discovered by CT scan and contrast administration through the port.

Another case of erosion into the bowel was reported by Holt et al. [6]: a woman with adenocarcinoma of the cecum with metastatic disease to the left ovary and invading into the sigmoid underwent right hemicolectomy and resection of the involved ovary and sigmoid. At the time, a Tenckhoff catheter was placed in the left abdomen. Following one session of chemotherapy, the patient developed fever and abdominal pain. Following contrast injection into the catheter, contrast was seen directly in the sigmoid colon indicating a fistula and the catheter was subsequently removed.

Our case is similar to the two cases described. First, the patient underwent a left hemicolectomy during the same laparotomy in which the catheter was placed. Similarly, the patients mentioned both had surgical procedures performed on the bowel with concurrent placement of the catheter. Second, the patient had a Bard catheter placed. This catheter has an implantable injection port and a Tenckhoff radiopaque catheter. Both of the cases discussed also used a Tenckhoff catheter, which is a fenestrated catheter. Third, the patient underwent several successful rounds of chemotherapy prior to manifestation of the catheter erosion into the bowel.

Several cases including those described by Wakefield et al. and Davidson et al. [4] have shown that rates of bowel injury and infection increased when the catheter was placed or was already in place at the time of bowel surgery. The catheter may be in close enough proximity to the surgical site that it disrupts the inflammatory process, leading to dehiscence [6]. Rates of this particular complication can be improved by performing the catheter insertion at a separate time from any procedure being performed on the bowel. The catheter should be placed in a separate laparotomy under direct vision with the tip of the catheter placed far away from the anastomotic or surgical site [6]. In this particular case, however, the catheter tip was not placed in the vicinity of the anastomotic site. It is unclear at this time how a fenestrated or Tenckhoff catheter may contribute to erosion into the bowel.

## 4. Conclusion

With the rising use of IP chemotherapy for the treatment of ovarian and colon carcinoma, we should continue to investigate the complications and their causes to reduce additional patient morbidity and mortality.
